# The current state of MRI-based radiomics in pituitary adenoma: promising but challenging

**DOI:** 10.3389/fendo.2024.1426781

**Published:** 2024-09-20

**Authors:** Baoping Zheng, Zhen Zhao, Pingping Zheng, Qiang Liu, Shuang Li, Xiaobing Jiang, Xing Huang, Youfan Ye, Haijun Wang

**Affiliations:** ^1^ Department of Neurosurgery, Union Hospital, Tongji Medical College, Huazhong University of Science and Technology, Wuhan, China; ^2^ Department of Neurosurgery, People’s Hospital of Biyang County, Zhumadian, China; ^3^ Department of Ophthalmology, Union Hospital, Tongji Medical College, Huazhong University of Science and Technology, Wuhan, China

**Keywords:** Pit-NET, pituitary adenoma, radiomics, textual analysis, machine learning, biomarkers, neuroimaging

## Abstract

In the clinical diagnosis and treatment of pituitary adenomas, MRI plays a crucial role. However, traditional manual interpretations are plagued by inter-observer variability and limitations in recognizing details. Radiomics, based on MRI, facilitates quantitative analysis by extracting high-throughput data from images. This approach elucidates correlations between imaging features and pituitary tumor characteristics, thereby establishing imaging biomarkers. Recent studies have demonstrated the extensive application of radiomics in differential diagnosis, subtype identification, consistency evaluation, invasiveness assessment, and treatment response in pituitary adenomas. This review succinctly presents the general workflow of radiomics, reviews pertinent literature with a summary table, and provides a comparative analysis with traditional methods. We further elucidate the connections between radiological features and biological findings in the field of pituitary adenoma. While promising, the clinical application of radiomics still has a considerable distance to traverse, considering the issues with reproducibility of imaging features and the significant heterogeneity in pituitary adenoma patients.

## 1 Introduction

Pituitary adenoma (PA, also known as pituitary neuroendocrine tumor, [Pi-NET]) is a relatively common entity, accounting for about 10%-15% of primary intracranial tumors (1–3). Most of these tumors are benign and typically present with hormone hypersecretion syndromes and/or mass effect on critical structures; patients with these benign tumors also experience poor quality of life (1). Moreover, over 30% of PAs may be invasive and infiltrate surrounding structures, including the cavernous sinuses, bone, hypothalamus, and internal carotid (4). Even with the inclusion of multidisciplinary comprehensive treatment, there remains a tendency for frequent recurrence (5). The management of pituitary lesions presents a significant challenge due to the intricate nature of pituitary occupancy and the heterogeneity of pituitary adenoma subtypes, necessitating precision identification methods and individualized management strategies.

In addition to hormonal assays and biopsy, the diagnosis and treatment plan of PA are highly dependent on imaging, mainly on magnetic resonance imaging (MRI). In patients suspected of harboring a pituitary lesion, it is widely recognized to conduct MRI scanning first to non-invasively identify tumor type, as well as ascertain mass size and location (6–8). Additionally, long-term radiological follow-up can provide vital information regarding tumor progression and postoperative recurrence. MRI, with the advantages of high soft tissue contrast, no radiation, and multiplanar imaging capability, proves to be an indispensable tool (9). However, owing to the diverse classification of pituitary adenomas, the wide range of clinical manifestations, and the similarity in imaging characteristics, traditional manual interpretation of radiological images remains limited in clinical practice. The actual effectiveness of these qualitative diagnoses highly relies on the experience and expert knowledge of the neuroradiologists, introducing an inherent issue of unavoidable human errors. In contrast, the advantages of objective quantitative methods lie in their high time efficiency, strong repeatability, and enhanced capability to recognize intricate features (10). Here, radiomics has emerged in response to this need.

Radiomics refers to quantitatively capturing features from routine medical scans through data characterization algorithms, enabling the detection of subtle cues that are not discernible to the naked eye (11). These features are high-dimensional, mineable data, containing information reflective of tumor texture and heterogeneity (10). In recent years, radiomics has been applied in various brain tumors, such as meningiomas (12, 13), gliomas (14–16), and metastases (17, 18), and other CNS tumors (19–21). Yet, only a few studies have reported on radiomics analysis of PA.

The aim of this review is to examine the current application of MRI-based radiomics in the management of PA from a clinical perspective, in a way that even those not familiar with computer science could understand. Specifically, we provide a concise overview of the workflow in radiomics, and systematically summarize and organize the information from five perspectives: diagnosis, subtyping, aggressiveness, consistency and treatment response assessment, and provide comparative tables for reference. Finally, we discuss the future prospects and limitations of radiomics in the field of PA.

## 2 Overview of radiomic pipeline

Radiomics refers to the extraction and analysis of large amounts of advanced quantitative imaging features with high throughput from radiological images, which can be employed for diagnosis, prognosis assessment, and adjunctive selection of therapeutic modalities (10). Compared to conventional radiology, radiomics offers the benefit of being less subject to individual radiologist biases and can capture a lot more additional information. The steps involved vary across different studies, each tailored to specific objectives (10). The workflow of classic radiomics typically involves the following steps:

- Image acquisition and reconstruction: This first step involves obtaining raw data from various medical imaging modalities such as CT, MRI, PET, etc. Then the raw non-image-formative data undergo reconstruction into 2D or 3D image format. Unfortunately, standardization of these parameters in acquisition and image reconstruction is still lacking, necessitating the provision of error bars to improve the reliability.
- Region of Interest (ROI) identification/segmentation: In this step, areas of interest, usually tumors or other pathological lesions, are identified in the images. It is crucial as the subsequent feature data are generated from the segmented areas. This can be delineated manually by a neuroradiologist; while this method is effective in ensuring accuracy, the process can be somewhat cumbersome and is subject to significant inter-observer variability (22). Alternatively, automatic identification can rapidly process large datasets, significantly improving work efficiency but may compromise accuracy in complex cases (23, 24), and semi-automatic segmentation techniques may find a balance between the two approaches (25).
- Feature extraction: Radiomic features are extracted from identified regions of interest (ROIs) under expert supervision, using predefined algorithms to reveal the biological characteristics of the tumor. These features can typically be divided into four categories: i Shape features focus on the geometry and size of the ROIs, effectively reflecting morphological changes in tumors. ii Texture features describe the spatial distribution and arrangement of pixel values within the image, making them suitable for analyzing tumor heterogeneity (26). iii Histogram features are derived from the statistical distribution of pixel values, without considering their spatial relationships. iv By applying complex mathematical transforms and filters, high-order features convert image data into alternative forms, enabling the extraction of deeper, more intricate information (10). It’s important to note that semantic features are cumulative diagnostic expertise of radiologists, facilitating the interpretation of lesions in a biologically meaningful context. While they are not radiomic features, they are often compared with radiomic features for model evaluation.
- Feature selection: Given the vast number of features extracted, one way to decrease the propensity for model overfitting linked to the high-dimensional nature of the radiomic feature set is to select the most representative and top-ranking features. Eliminating irrelevant features is imperative, as their presence can obscure the significance of related features and negatively impact the predictive model’s performance. There are four common algorithmic approaches: i Filter Methods: Select features based on statistical criteria, such as variance thresholding to remove low-variance features. ii Wrapper Methods: Choose features based on model performance, like forward selection, which iteratively adds features that improve performance. iii Embedded Methods: Perform feature selection during model training, automatically identifying important features, such as least absolute shrinkage and selection operator (LASSO) regression using L1 regularization (27). iv Statistical Methods: Techniques like principal component analysis (PCA) reduce dimensionality by transforming high-dimensional data into a lower-dimensional space through linear combinations of the original features (28).
- Modeling: Once optimal features are identified, feed the data, the features of training set that are annotated with categorical labels, into the classification algorithms (classifier). The labels represent the categories of interest (e.g., invasive and non-invasive). It is noteworthy that the model can integrate not only radiomic features but also additional information, such as clinical, demographic, or genomic data (10).

**Figure 1:**
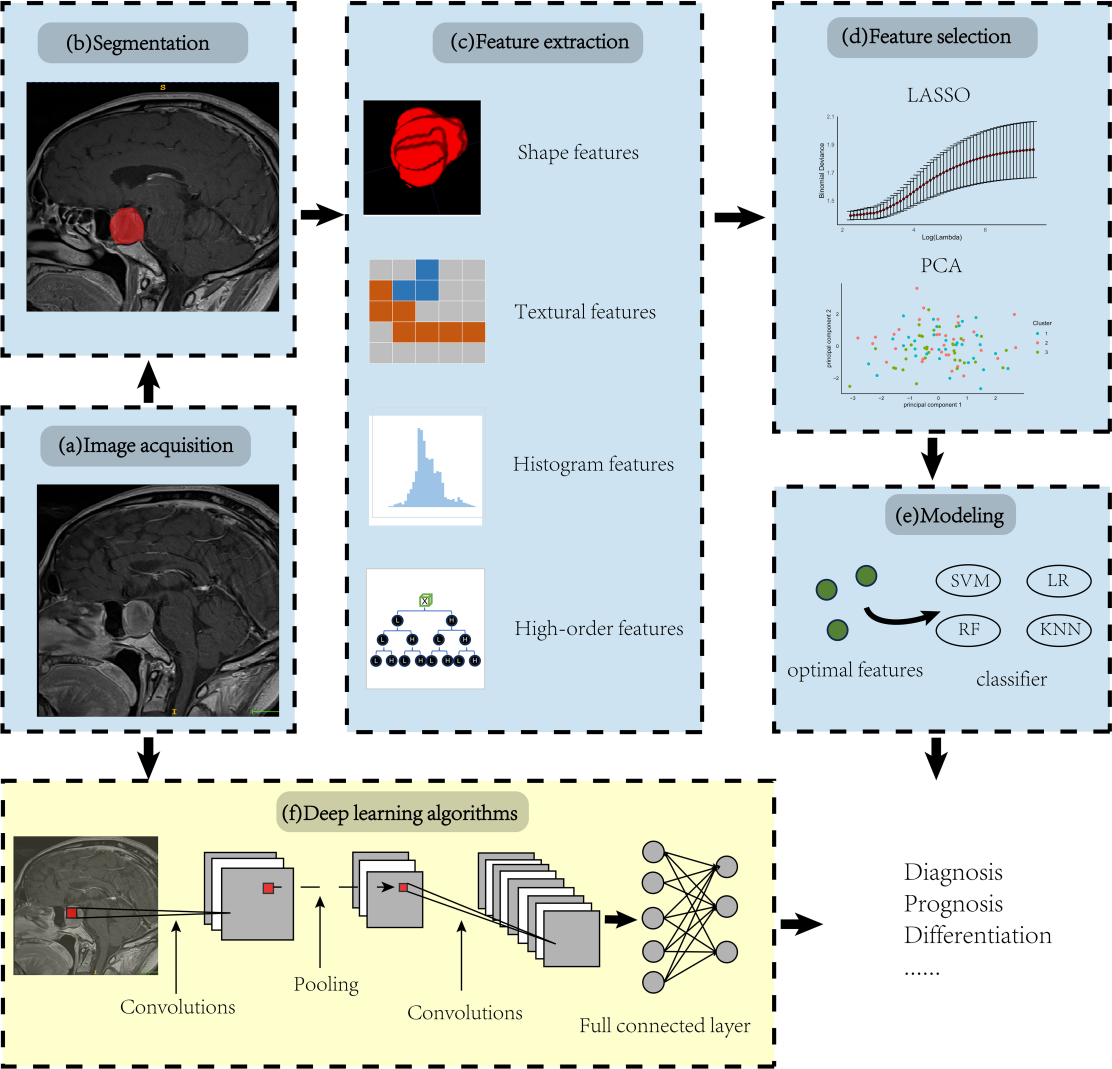
Overview of radiomic pipeline. Panels **(A-E)** illustrate the manual radiomics workflow, while panel **(F)** demonstrates the integration of a deep learning radiomics algorithm using a Convolutional Neural Network (CNN) as an example.

The core of radiomics is quantitative analysis of imaging features; thus, not all radiomics studies strictly adhere to the aforementioned processes. For instance, deep learning models, such as convolutional neural networks (CNN), learn not only the features but also how to map these features to the predicted outcomes, which directly integrate the processes of feature identification, selection, and even modeling across different layers (**Figure 1**).

## 3 Application in pituitary adenoma

### 3.1 Application in differential diagnosis

When lesions are situated in the sellar and suprasellar regions, it is often difficult to differentiate pituitary adenomas, craniopharyngiomas, meningiomas, Rathke cleft cysts, and inflammatory processes from one another based solely on MR imaging (29). Given the distinct surgical strategies and treatment protocols for each of these conditions, correct preoperative diagnosis of these lesions is clinically critical. The advancement of radiomics offers a new avenue to meet this challenge.

Zhang et al. (30) conducted a study where they retrospectively extracted qualitative MRI features and textual features from 126 patients diagnosed with pituitary adenoma (N = 63) or craniopharyngioma (N = 63). The results indicated that a radiomic feature from T1CE and two features from T2WI could act as independent diagnostic predictors. Besides, cystic change was the only independent diagnostic predictor among the image features, and histogram skewness, gray level co-occurrence matrix contrast (GLCM-Contrast) from the textual features extracted from T2WI was significantly associated with the macroscopic cystic change (p ≤ 0.001). This suggests that textual analysis may provide a microscopic perspective on the tissue heterogeneity of cystic changes. In another study conducted by the same team (31), they applied five feature-selection methods (distance correlation, random forest (RF), least absolute shrinkage and selection operator (LASSO), extreme gradient boosting (XGboost), and gradient boosting decision tree (GBDT)) and nine machine-learning classifiers (linear discriminant analysis (LDA), support vector machine (SVM), random forest (RF), adaptive boosting (AdaBoost), k-nearest neighbors (KNN), Gaussian naive Bayes (GaussianNB), logistic regression (LR), gradient boosting decision tree (GBDT), and decision tree (DT)) for discrimination of sellar masses (including craniopharyngioma, pituitary adenoma, Rathke cleft cyst, and meningioma). They found that LASSO stands out as an exceptional feature-selection technique and LASSO + LDA model demonstrated superior aggregate performance, achieving an area under the curve (AUC) exceeding 0.80 across all training and testing cohorts. It’s noteworthy that the average AUC of the general radiologists in the differentiation among pituitary macroadenoma, craniopharyngioma, and Rathke cleft cyst is 0.876, but for the neuroradiologists it’s 0.952 (32). This indicates that radiomic models that solely rely on cues from medical imaging predominantly play a role in assisting diagnosis, yet there remains a discernible gap in diagnostic efficacy compared to experienced neuroradiologists.

Wang et al. (33) aimed to explore the diagnosis value of MRI-based radiomics model for the classification of cystic pituitary adenoma and Rathke cleft cyst. They developed six models, three of them based on single sequence radiomic features: T1WI model, T2WI model, and postcontrast T1WI model, as well as multiparametric radiomics models, semantic models, and the combined radiomics and semantic model, and compared their diagnostic efficacy. The findings published indicated an enhancement in the performance of the radiomics model upon the addition of semantic features. Compared to the radiologist mentioned in the text, the machine-learning model based on radiomics exhibits superior diagnostic performance.

Regrettably, none of the three studies incorporated clinical information in the establishment of a diagnostic model. Zhao et al. (34) formulated and validated a diagnostic nomogram for classifying Cystic-Solid pituitary adenoma from craniopharyngioma, which integrates radiomic signatures with hematological parameters, encompassing four elements: radiomics signature, patient age, white blood cell (WBC) count, and fibrinogen (FIB) levels. In both the training and testing sets, the radiomic-clinical nomogram notably outperformed the radiomics-only model, evidenced by p-values of 0.031 and 0.038, respectively.

Analogous to the clinical manifestations of pituitary adenomas, hypophysitis may present as a mass effect or aberrant hormone secretion. Although a novel radiological approach introduced by Gutemberg et al. significantly improved the differential diagnosis of hypophysitis and non-functioning pituitary adenoma (NFPA), the diagnostic criterion still possesses a certain degree of misdiagnosis (35–37). This underscores the necessity for more precise methods to differentiate between these two conditions. Sahin et al. (38) manually depicted ROI images from coronal and sagittal planes in a three-dimensional (3D) fashion and extracted textual features from each T2 weighted coronal, T1CE coronal, and T1CE sagittal MRI. Top 3 features, those that could differentiate the two lesions, were employed to build a machine learning diagnostic model. The linear SVM classifier showed the highest performance among all classifiers, with AUC of 0.91.

Approximately 40% of all pituitary adenomas are microadenomas (< 10 mm in diameter), that may remain undetectable by radiologist, even if experienced experts and advanced instrumentation including dynamic contrast enhanced imaging techniques are applied (39, 40). This phenomenon is particularly pronounced in adrenocorticotropic hormone (ACTH)-secreting adenomas, although selective inferior petrosal sinus sampling (IPSS) serves as the gold standard for MRI-negative ACTH adenoma, clinicians remain cautious in its use due to its invasive nature (7). Recognizing this challenge, Li et al. (41) first attempt to propose a pituitary microadenoma (PM) diagnosis system from MRI by CNN. The algorithm’s strength is its ability to automatically identify the ROI area and features extraction, achieving a diagnostic accuracy of 96.5% in an independent validation set, matching the performance of radiologists with over ten years of experience in the field. Most importantly, the authors have launched a user-friendly tool, requiring only the upload of a patient’s T1CE images. The algorithm provides diagnostic results within 1-2 seconds. This innovation aims to reduce the workload of radiologists and assist clinicians in making diagnoses. A summary of the diagnostic-related literature is presented in **Table 1**.

**Table 1:** Application in differential diagnosis.

Author	Year	Differentiation Type	NO. of Patients	MRI sequences	Machine learning/statistical method	outcomes
Zhang (30)	2020	pituitary adenoma VS craniopharyngioma	126 (PAs = 63, craniopharyngiomas = 63)	T1CE, T2	Binary logistic regression analyses	Three textual features were able to act as independent diagnostic predictors, and the presence of cystic change was significantly linked to HISTO-Skewness and GLCM-Contrast.
Zhang (31)	2020	pituitary adenoma vs. craniopharyngioma, meningioma vs craniopharyngioma, and pituitary adenoma vs Rathke cleft cyst	235 (craniopharyngiomas = 63, meningiomas = 64, PAs = 68 and Rathke cleft cysts = 40)	T1CE	LDA, SVM, RF, AdaBoost, KNN, GaussianNB, LR, GBDT, DT	The integration of LASSO for feature selection and LDA for classification appeared to be the optimal model for discrimination of lesions located in the anterior skull base among the 45 diagnostic models, with AUC of over 0.80 in all the three comparison groups.
Zhao (34)	2021	Qian A8(101)	272 (cystic-solid PAs = 201, craniopharyngiomas = 61)	T1CE, T2	logistic regression, Ridge classifier, SGD classifier, Linear SVM, MLP	The radiomic-clinical nomogram demonstrated significantly better performance than the radiomics model, both in the training set (p=0.031) and the test set (p=0.038).
Wang (33)	2021	cystic pituitary adenoma VS Rathke cleft cyst	215 (cystic PAs = 105, Rathke cleft cysts = 110)	T1CE, T1, T2	SVM, ANN, AdaBoost, RF	The integrated radiomics and semantic model with ANN classifier achieving the highest diagnostic performance with a mean AUC of 0.924, better than the radiologist
Sahin (38)	2022	non-functional pituitary adenoma VS hypophysitis	34 (NFPAs = 17, hypophysitises = 17)	T1CE, T2	LDA, fine, medium and coarse decision trees, KNN, SVM, naive Bayes, ensemble classifiers	Linear SVM classifier based on top 3 features, those extracted in a 3D fashion, showed feasible performance in discriminating hypophysitis from NFPAs (AUC=0.91).
Li (41)	2021	diagnose pituitary microadenoma (PM)	1520 (PMs = 556, controls subjects = 964)	T1CE	CNN	The PM-CAD system achieves a 96.5% diagnostic accuracy, comparable to experienced radiology experts.
Qian (101)	2020	pituitary adenoma VS non-pituitary adenoma	149 (PAs = 84, control subjects = 65)	T1, T2	CNN	The CNN algorithm based on multiple MR images exhibits exceptional performance with an accuracy of 96.97%.

### 3.2 Application in distinguishing subtypes

Differentiated therapeutic strategies are necessitated by the diverse types of pituitary adenomas. Management of functional pituitary adenomas primarily focuses on controlling hormone hypersecretion, whereas the management of non-functional pituitary adenomas centers on addressing tumor growth and mass effects (7). Laboratory tests can quickly and accurately differentiate these conditions. However, other causes, such as medication side effects, hypothyroidism, or the stalk effect from other masses, can also result in abnormal hormone levels (42). The hook effect may present misleadingly low values (42, 43). In cases where confounding factors impact laboratory tests or results are unclear, radiomics offers an alternative approach to confirm diagnoses or supplement laboratory findings. Furthermore, the pathological classification of PAs relies on immunohistochemistry and even electron microscopy (3). Patients who cannot undergo tissue biopsy cannot benefit from subtype-specific treatments. Recently, several investigations have aimed to clarify the relationship between radiomic features and histopathological characteristics.

Sanei Taheri et al. (44) applied first and second-order histogram analysis on diffusion-weighted images (DWI) of 32 patients with macroadenoma and reported that smoothness and uniformity are proposed as indicators for non-functional tumors, whereas the 75th percentile is deemed more suitable for the diagnosis of functional tumors. DWI is seldom used as a standard procedure for imaging the sellar region; thus, a method that utilizes routine MRI sequences is more likely to be widely adopted in clinical practice. Carlo et al. (45) selected 28 texture features from coronal T2WI images, and through the construction of models using J48, multinomial logistic regression, and K-nearest neighbor machine learning algorithms, they were able to accurately classify these adenomas into functional and non-functional subtypes, achieving similarly favorable discrimination outcomes as Sanei Taheir. Li et al. (46) introduced the methodology of transfer learning to build a CNN classification model and used the pre-trained segmentation model to derive radiomic features from 3D MRI images. The multi-view (axial, sagittal and coronal view) automated segmentation model is capable of accurately identifying the region of PAs, consistently achieving Dice scores above 0.8 across both validation sets and a test dataset. By integrating the transfer-learning method and the attention mechanism, the classification model was able to predict functioning and non-functioning PAs with an AUC equal to 0.848 in the test set.

In the 5th Edition of the WHO Classification of Endocrine and Neuroendocrine Tumor, PAs are recommended to be classified based on cell lineage as determined firstly by expression of transcription factors (3). Peng et al. (47) investigated the correlation between pituitary transcription factors and MRI radiomic features and found that features extracted from coronal T2WI could provide more information than other sequences in classification of PAs. Additionally, their SVM model functioned as a multiclass classifier distinguishing it from the majority of similar studies that engaged in binary classification. Another group analyzed the T2WI cohort included 176 patients with seven different subtypes of pituitary adenomas and built a multivariable diagnostic prediction model to differentiate the hormone secretion profile of pituitary adenomas. It’s worth noting that all models exhibited an AUC greater than 0.85, except for the one distinguishing PHA (pluri-hormonal secreting adenomas), which had an AUC of 0.74. This could be attributed to the various cell types and significant tissue heterogeneity associated with PHA (48).

In comparison to densely granulated (DG) somatotroph adenomas, sparsely granulated (SG) adenomas exhibit a greater size, enhanced invasiveness, and a less favorable prognosis. Granulation pattern were only recognized via electron microscopy imaging or cytokeratin immunohistochemistry according to the 2017 WHO Classification (49), neither of the two methods can be applied preoperatively. This has also been noted by researchers, Park et al. (50)showed that a radiomics model based on T2WI and T1CE with excellent performance distinguishing granulation pattern of GH-PA patients (AUC =0.834). Likewise, Liu et al. (51) validated the efficacy of features from T2WI with an AUC of 0.823, but surprisingly T1WI signatures achieved highest performance with an AUC of 0.918, even better than radiomics signatures combined the texture features of T1WI and T1CE (AUC=0.908). In addition, the authors noted that variations in ROI delineation would impact the outcome of radiomics, indicating that it obviously yields superior outcomes when performing tumor segmentation by excluding the cystic/necrotic portion instead of incorporating the entire tumor region.

Due to the absence of hormone secretion in non-functioning adenomas (NFPAs), differentiating NFPA subtypes proves more challenging than subtype differentiation based solely on preoperative hormone secretion. Zhang et al. (52) demonstrated that radiomics is an effective tool in differentiating between null cell adenomas (NCAs) and other NFPA subtypes, with an AUC equal to 0.804 obtained from T1WI in the test set. The model built on selected T1CE features demonstrates strong performance in the training set, yet exhibits a lack of discriminative ability in the test set, indicating a potential risk of overfitting. The variance in uptake rates of the contrast agent Gd-DTPA among patients may affect the T1CE texture features and the model might have overly learned the data characteristics in the training set, including specific texture features caused by varying renewal rates, which may not be applicable or prominent in the test set leading to poor generalization. Besides, they found that the inclusion of T1CE features did not provide any extra contribution to predicting NCAs compared to T1WI features alone, therefore T1WI was chosen to be the best sequence.

Silent corticotroph adenomas (SCAs) had a higher propensity for cavernous sinus invasion, along with increased rates of progression and recurrence (53, 54). The evaluation of new identifying markers, along with the exploration of emerging imaging, is essential for effectively addressing the needs of this distinct patient population. Rui et al. (55) has made efforts in this regard and their ensemble model surpassed the existing clinical approach in predicting patients with SCAs among NFPAs through the application of radiomics. It’s worth mentioning that their study utilized super-learner algorithms, employing a voting mechanism which incorporated the predictions from other individual classifiers. The ensemble model, trained with both semantic and radiomic features, indeed yielded excellent results (AUC of 0.926 and accuracy of 0.867). In the context of clinical practice, physicians pay attention to age, gender, invasiveness, and cystic change when prognosticating SCAs in patients who are hormone-negative (54). However, in the ensemble algorithm, radiomic prediction emerged as the most critical risk factor, followed by clinical-associated features mentioned earlier. In another study for the preoperative diagnosis of silent corticotroph adenomas, Wang et al. (56) demonstrated that the linear SVM classifier exhibited the best performance, achieving the highest AUC value of 0.931 for the internal dataset and 0.937 for the external dataset. To enhance model interpretability and rank the contributions of features, the authors introduced an algorithm called SHAP (Shapley Additive exPlanations) and proved radiomics-based features extracted from T1CE and T2WI played a critical role in the machine-learning model, with their impact on predictions second only to multiple microcysts and age. A more detailed summary of radiomic research applied in subtype differentiation is presented in **Table 2**.

**Table 2:** Application in distinguishing subtypes.

Author	Year	Differentiation Type	NO. of Patients	MRI sequences	Machine learning/statistical method	outcomes
S. Taheri (44)	2019	Distinguishing functional from non-functional pituitary macroadenomas	32 (functional adenomas=10, NFPAs=22)	DWI	Mann-Whitney U test	Histogram-extracted smoothness, uniformity and 75th percentile based on DWI were able to differentiate FPA from NFPA. Cut-off points of FSOH features were proposed.
Carlo (45)	2020	Distinguishing functional from non-functional Pituitary macroadenomas	50 (functional adenomas=25, NFPAs=25)	T2	J48, a multinomial LR, KNN	Multinomial logistic regression and K nearest neighbor achieved accuracies beyond 92.0% and the AUC of ROC till 98.4%.
Peng (47)	2020	Classified based on transcription factors	235 (TPIT lineage tumors=55, PIT1 lineage tumors=110 and SF-1 lineage tumors=70)	T1, T2, T1CE	SVM, KNN, Naïve Bayes models	The SVM model exhibited optimal classification performance and T2WIs performed better than T1, T1CE sequences.
Li (46)	2021	Distinguishing Functional from non-functional Pituitary adenomas	185 (functional adenomas=125, NFPAs=60)	T1, T2, T1CE, FLAIR	CNN	The CNN-based automatic segmentation model effectively handles 3D MRI segmentation tasks, achieving a Dice coefficient of up to 0.818. The CNN-based classification model also exhibits strong performance, with an AUC of 0.848.
Baysal (48)	2022	Classified based on hormone secretion profiles	130 (NFPAs=19, GHs=21, PRLs=64, ACTHs=6, PHAs=6, FSA&LHs=8, and TSHs=6)	T2	ANN	The performance of ANN in distinguishing prolactinomas from other adenomas is the highest (AUC=0.95), while the model for distinguishing PHAs exhibits the lowest AUC (AUC=0.74). The AUC values for the other 4 ANN were >0.85.
Park (50)	2020	Classified based on granulation pattern of growth hormone secreting pituitary adenoma patients	69 (DGs = 50, SGs = 19)	T2, T1CE	generalized linear model	The radiomics model demonstrated better performance (AUC=0.834)than qualitative assessment (AUC=0.597)or T2 signal intensity evaluation identifying granulation patterns(AUC=0.647).
Liu (51)	2021	Classified based on granulation pattern of growth hormone secreting pituitary adenoma patients	49 (DGs = 24, SGs = 25)	T1, T2, T1CE	LASSO LR	T1WI signatures achieved the highest diagnostic efficacy with an AUC of 0.918, better than the combined radiomics signatures(AUC=0.908), but the decision curve analysis indicates a more pronounced benefit of the latter approach.
Zhang (52)	2018	Distinguishing null cell adenomas (NCAs) from other NFPA subtypes	112 (NCAs=46, other NFPA subtypes=66)	T1, T1CE	radial basis function SVM	The T1 predictive model was selected as the ultimate model for distinguishing NCAs from other NFPA subtypes, with AUC=0.8042.
Rui (55)	2022	Distinguishing silent corticotroph adenomas (SCAs) from other NFPA subtypes	302 (SCAs=146, other NFPA subtypes=166)	T1, T2, T1CE	penalized logistic regression, SVM, LDA, RF, gradient boosting machine, neural network, ensemble algorithm	The ensemble model, trained with both semantic and radiomic features produced excellent results (AUC of 0.926). T2WI features outperformed other single MR modalities.
Wang (56)	2023	Distinguishing silent corticotroph adenomas (SCAs) from other NFPA subtypes	295 (SCAs=78, other NFPA subtypes=217)	T1, T2, T1CE	Elasticnet, Linear SVM, RF, ET, KNN, DT, GBDT, AdaBoost, MLP, XGBoost	The Linear SVM classifier exhibited the best performance and features derived from T1CE and T2 imaging played a pivotal role in the predictive analysis.
Galm (90)	2020	Distinguish NFPAs from somatotroph adenomas	263(somatotroph adenomas=85, NFPAs=78)	T1	LR	Kurtosis showed strong diagnostic accuracy with an AUC of 0.7771 in distinguishing between the two.

### 3.3 Applications in aggressiveness assessment

Approximately 40% of PAs present an aggressive behavior (57). Currently, there is no solitary prognostic parameter capable of decisively determining the risk of growth or malignant progression (58). Assessing tumor proliferation through measures such as mitotic count and/or Ki-67 labeling index, or evaluating tumor invasion into surrounding tissues, could hold significance on a case-by-case basis, given their correlation with more aggressive tumor behavior (59).

Although the 2017 and the latest 2022 WHO classifications of PA have abandoned Ki-67 index as a grading criterion, Ki-67 index of >3% is still an indispensable prognostic marker in the assessment of tumor aggressiveness (3, 5, 60). Numerous studies have demonstrated the efficacy of radiomics in accurately predicting the Ki-67 index across various tumor types, such as lung cancer (61), breast cancer (62),and glioma (63).Recent research has also validated the potential of radiomics in prognosticating the Ki-67 index in pituitary tumor tissues, underscoring its growing relevance in the field of oncological biomarker identification and prognostic assessment. Ugga et al. (64) were the first to propose that the application of radiomics is effective in predicting the Ki-67 proliferation index of pituitary adenomas. They correlated textural analysis on preoperative T2WI of 89 patients with postoperative pathology and reported an accuracy of 91.67% in predicting Ki-67 proliferation index class for macroadenoma.

In a multicenter study involving 163 patients with acromegaly, based on T1, T2, and T1CE images, Fan et al. (65) reported that the radiomics signature achieved a satisfactory result in predicting the Ki-67 index with AUC values of 0.96 (95% confidence interval [CI]: 0.95-0.98) in the primary cohort and 0.89 (95% CI: 0.87-0.91) in the validation cohort, demonstrating superior performance over clinical model in both the cohorts. However, despite the superior clinical utility of the radiomics nomogram, as evidenced by the decision curve analysis (DCA) curve, the results of the DeLong test indicate no significant difference in performance between the radiomics signature and the radiomics nomogram.

When tumor tissues invade surrounding structures, it is more difficult to surgically remove invasive pituitary adenomas compared with noninvasive pituitary adenomas. The definition of invasive pituitary tumors before surgery, as accepted today, is largely based on imaging data (66). Distinguishing invasion from compression is challenging, often not visibly detectable. For an extended period, the Knosp classification has been the acknowledged standard in radiological identification of invasiveness. Within this framework, Grade 0 indicates no invasion of the cavernous sinus, while Grade 4 denotes definite invasion, correlating with a 100% incidence of surgical and histological invasion (67). However, Grades 2 and 3 represent a “gray area” where the level of invasiveness is not distinctly clear. Niu et al. (68) established a radiomics nomogram for the personalized assessment of cavernous sinus invasion in 194 patients with PAs, specifically those with Knosp grades two or three. Incorporating the radiomics signature from the T1CE images, Knosp grade, periarterial enhancement, and inferolateral venous compartment obliteration, the radiomics nomogram yielded an AUC of 0.899 in the training set and 0.871 in the test set, even performing better than the clinico-radiological model and radiomics models based on T1CE, T2, and T1CE&T2 images (p = 0.021 and p= 0.035 in the training and test sets, respectively). Liu et al. (69) elucidated the invasiveness of pituitary adenomas based on texture analysis of dynamic contrast-enhanced MRI (DCE-MRI), interpreting the results from the perspective of tumor angiogenesis and microvascular permeability. The morphological features results show that the greater the volume and the more irregular the shape, the higher the likelihood of the PM being aggressive. A plausible explanation for this phenomenon could be the aberrant angiogenesis occurring within aggressive tissues, leading to a relatively irregular growth rate and heterogeneous expansion. Similar results have also been corroborated by Wang and colleagues (70). They ultimately selected 2 shape-related and 2 textural optimal features from 399 patients for the construction of a diagnostic model. In the training cohort, the morphological features-based model demonstrated an AUC of 0.86, compared to 0.75 for the textural features-based model. Combining both radiomic features with Knosp grades was shown to be more effective. The AUC of composite parameter model reached 0.935 for the test set. Moreover, an association was discerned between these radiomic features derived from volumetric T1CE images and the high Ki-67 labeling index, high mitotic count, and positive p53 expression within pituitary adenomas (all p values < 0.05). Zhang et al. (71) developed a model to predict highly invasive pituitary adenomas using preoperative T1CE images and linear SVM algorithms. Employing filter transformation with the original features enhanced the model’s ability to utilize refined texture features, resulting in an AUC of 0.73 in the validation set. Literature related to aggressiveness in radiomics is summarized in **Table 3**.

**Table 3:** Application aggressiveness assessment.

Author	Year	Differentiation Type	NO. of Patients	MRI sequences	Machine learning/statistical method	outcomes
Ugga (64)	2019	predict the Ki-67 index	89 (low Ki-67 = 59, high Ki-67 = 30)	T2	KNN	KNN classifier on texture-derived features proved to be an effective tool in the prediction pituitary macroadenomas’ Ki-67 index and the four selected features all showed very good inter-observer reproducibility (ICC ≥0.85).
Fan (65)	2020	predict the Ki-67 index	138 (low Ki-67 = 56, high Ki-67 = 82)	T1, T2, T1CE	SVM	Radiomics nomogram achieved an area under the curve (AUC) value of 0.91 in the validation cohort, demonstrating comparable performance to radiomics signature.
Wang (70)	2023	predict aggressiveness	246 (aggressive = 84, non-aggressive = 162)	T1CE	Stepwise LR	Two shape-related and two textural features were significantly associated with a high Ki-67 labeling index (Ki-67 LI ≥ 3%), high mitotic counts, and positive p53 expression.
Liu (69)	2020	predict aggressiveness	50 (aggressive=32, non-aggressive=18)	dynamic contrast-enhanced MRI (DCE-MRI)	LR	Texture analysis based on DCE-MRI was able to assess the vascular heterogeneity and aggressiveness of PM.
Zhang (71)	2022	predict aggressiveness	196 (aggressive=95, non-aggressive=81)	T1CE	Linear SVM	The radiomics model based on preoperative T1CE images could predict cavernous sinus invasion.
Niu (68)	2019	predict invasiveness	194 (invasive=82, non-invasive=112)	T1CE, T2	Linear SVM	In conjunction with clinical risk factors, the T1CE radiomics signature was chosen to construct a diagnostic nomogram, achieving an AUC of 0.871.

### 3.4 Applications in consistency assessment

Consistency is one of the most important risk factors influencing the complexity of the surgical procedure. It is easier to treat softer tumors through a transsphenoidal approach, while firmer, fibrous tumors might necessitate more aggressive surgical techniques or instruments (72). Accurately predicting this consistency preoperatively can aid in designing an effective surgical plan, reduce the need for multistage surgeries, and improve patient prognosis. The collagen content is a critical factor determining the texture of PA, and it ultimately affects the performance of MRI imaging (73). However, Bahuleyan and colleagues (74) indicated that the signal intensity of MR imaging alone does not reliably predict the consistency of pituitary macroadenoma. This unpredictability underscores the necessity for more sophisticated diagnostic tools.

Evidence from multiple studies indicates that radiomics analysis offers significant potential in enhancing diagnostic accuracy. R. Cuocolo et al. (75) established a methodology based on texture analysis of T2-weighted radiomics, employing machine Extra Tree classifier. In the study involving 89 patients (comprising 68 soft and 21 fibrous macroadenomas), a subset of 14 textual features was utilized. The final model exhibited an accuracy of 93%, a sensitivity of 100%, and a specificity of 87% in the test set. The AUC value was recorded at 0.99. Similarly, Zeynalova et al. (76) employed histogram radiomic features to describe tumor consistency. Their ANN model, based on the extraction of tumor texture features, performed better than conventional SIR evaluation (p= 0.021). These findings highlight the superiority of radiomics over traditional methods. Mendi et al. (77) extracted 206 ROIs from 52 patients who underwent surgical excision of pituitary adenomas for classifying soft consistency from hard. The diagnostic performance of SVM and RFC was as follows: sensitivity = 95.580% and 92.950%, specificity = 83.670% and 88.420%, area under the curve = 0.956 and 0.904, respectively. In a study focused on patients with acromegaly, by combining radiomic features and clinical parameters, Fan et al. (78) could more accurately determine the consistency of PA than using clinical characteristics alone. The radiomics model achieved favorable discriminability in the multicenter prospective validation, which was underscored by an AUC of 0.89 in the ROC analysis. Importantly, the Gray Level Co-occurrence Matrix (GLCM), a statistical method for analyzing image textures, was identified as the most pertinent imaging feature.

Unlike previous work focused on feature extraction from single sequences, Wan et al. (79) conducted a comprehensive set of radiomic features using T1, T2, enhanced T2, and their combinations, and systematically evaluated the utility of radiomics analysis of multiparametric MRI in assessing the consistency of PMA. The results showed that the radiomics model built on combined T1WI/T1CE/T2WI demonstrated the best performance with an AUC of 0.90 (95% CI: 0.87–0.92), an accuracy of 0.87, and a sensitivity of 0.83. Interestingly, they applied automated 3D segmentation instead of manual 2D, providing better visualization of tumor structures, which could add value to individualized treatment. The summary of articles related to the application of radiomics in consistency assessment is presented in **Table 4**.

**Table 4:** Applications in consistency assessment.

Author	Year	Definition of consistency	NO. of Patients	MRI sequences	Machine learning/statistical method	outcomes
R. Cuocolo (75)	2020	based on surgical features	89 (soft=68, hard=21	T2	ET	The ET model demonstrated high accuracy in classifying soft and fibrous pituitary macroadenomas.
Wan (79)	2022	based on surgical features	156 (soft=104, hard=52)	T1, T1CE, T2	RF, SVM	The RF classifier built on combined T1WI/T1CE/T2WI yielded the best performance (AUC = 0.90).
Mendi (77)	2023	based on surgical features	52*	T1, T2	SVM, RF	The performance of SVM based on T2W is optimal with an AUC of 0.956.
Zeynalova (76)	2019	based on surgical and histopathological findings	55 (soft=42, hard=13)	T1, T1CE, T2	ANN	The ML-based histogram analysis performed better than SIR evaluation in predicting PMA consistency (p = 0.021).
Fan (78)	2019	based on surgical features	158 (soft=100, hard=58)	T1, T2, T1CE	SVM	Incorporating clinical characteristics significantly augments the accuracy of radiomics models in forecasting tumor consistency among patients with acromegaly.
S. Taheri (44)	2019	based on collagen content	32 (soft =28 and hard=4)	DWI	Kruskal-Wallis test	First and second-order histogram features on DWI are not applicable for differentiation of high-content collagen macroadenomas from low-content types.

*The relevant data is not available.

### 3.5 Application in assessing treatment response

In the context of functioning adenomas, the postoperative basal hormone level emerges as the paramount predictor for the likelihood of recurrence (80, 81). However, for NFPA, the identification of a singular, persuasive predictive factor for recurrence remains elusive (81). Texture analysis revealed that NFPA patients with mean pixel intensity above the median had a lower recurrence or progression risk, with a hazard ratio of 0.44 (95% CI: 0.21-0.94, P = 0.034), compared to those below the median (82). This finding underscores the potential of radiomic analysis in risk stratification. Zhang et al. (83) applied an automatic segmentation approach and built a radiomics model to predict clinical outcomes in NFPAs, presented in terms of progression-free survival (PFS). They found that patients with higher SVM score tended to exhibit poorer PFS. The SVM score based on 3 selective radiomic features achieved an AUC of 0.87 in differentiation of early progression/recurrence. Furthermore, Shen and colleagues (84) attempted to elucidate the role of radiomics in predicting the regrowth of postoperative residual NFPA. They constructed logistic regression models utilizing both pre-operative and post-operative characteristics derived from individual sequences (T1WI, T1CE, and T2WI) as well as combined sequences (T1WI&T1CE, T1WI&T2WI, and T1CE&T2WI) and concluded that T1WI&T1CE was the optimal sequence to construct the radiomic score, which incorporates various radiomic features and their respective weights. Besides, the study emphasizes that features derived from postoperative images are significant references for researching residual tumors. Incorporating parameters such as Knosp grade and tumor volume doubling time can aid in optimizing the predictive accuracy of radiomic models.

As a pituitary tumor enlarges, it may extend superiorly and exert pressure on the optic chiasm, typically leading to a distinctive pattern of visual field loss termed bitemporal hemianopsia. While the degree of visual field recovery varies greatly, even if the changes seems to fit the expectation of the decompression procedure. Previous research has demonstrated that the MRI-based radiological characteristics of the optic nerve are associated with visual function and outcome in the patients with optic neuritis (85, 86). Radiomic features may serve as predictive markers for distinguishing between reversible axonal damage and permanent injury. Zhang et al. (87) successfully predicted visual recovery using preoperative T2WI in compressive lesions caused by pituitary tumors, radiomic models using three machine learning algorithms all achieved AUCs over 0.750. Subsequently, the same team further collected a set of consecutive images acquired through the optic chiasm before and after endoscopic endonasal transsphenoidal surgery, and analyzed the dynamic radiomics feature changes (or termed delta-radiomics) to predict visual outcome (88). Surprisingly, while morphological alterations of the optic chiasm after surgical decompression showed no significant differences between the recovery and non-recovery groups, one delta-radiomic feature provided predictive value, with an AUC of only 0.653. The final delta-radiomics model showed an AUC of 0.811 in independent testing data and after age correction, the model demonstrated an improved AUC value, reaching 0.841.

In addition to surgical intervention, pharmacological therapy is available for two specific Pit-NET subtypes, i.e., dopamine agonists for PRL Pit-NETs, somatostatin analogs (SAs) for GH Pit-NETs. Identifying prolactinomas that are resistant to dopamine agonists (DAs) early is vital, as it prevents patients from undergoing an extended period of ineffective therapy before exploring other treatment options. Park et al. (89) reported that conventional imaging parameters such as cystic/hemorrhagic change or T2 relative signal intensity showed no notable differences between the DA responders and non-responders; while the subtle distinctions could be captured via radiomic features and aid in assessing DA response of patients with PRL adenomas. Utilizing a soft voting ensemble classifier that amalgamated predictions from five distinct models, the optimal performance was observed in the test set. It achieved an AUC of 0.81 (95% confidence interval, 0.67-0.96), an accuracy of 77.8%, a sensitivity of 78.6%, and a specificity of 77.3%. In patients with acromegaly, Galm, B. P. et al. (90) discovered that MRI texture features of T1WI, particularly maximum pixel intensity, were significantly associated (p = 0.0143) with the normalization of IGF-I levels following SA therapy, but the association weakened after adjusting for granulation density through logistic regression. In addition to analysis of the original images, Kocak, B. et al. (91) applied Laplacian of Gaussian (LoG) filtering and wavelet transformation to T2WI data to extract higher-order features. The KNN classifier built on those features outperformed the quantitative and qualitative assessments of relative signal intensity, as well as the evaluation of immunohistochemical granulation patterns in terms of predictive accuracy.

Correspondingly, patients undergoing radiation therapy may also encounter side effects such as radiation encephalitis, cognitive disabilities, and in rare cases, secondary tumors (92–94). Thus, it’s crucial to select the most appropriate patients who are sensitive to this therapy. Fan et al. (95) demonstrated that MRI-based radiomics would provide an effective non-invasive tool for radiotherapeutic response prediction in patients with acromegaly. Six selected features achieved statistically significance differences between the remission group and non-remission group (P = 0.0005–0.0494) and radiomics signature built on these features performed better than the one based on five pre-radiotherapy clinical characteristics. The relevant literature is displayed in **Table 5**.

**Table 5:** Application in assessing treatment response.

Author	Year	Aim of study	NO. of Patients	MRI sequences	Machine learning/statistical method	outcomes
Galm (82)	2018	Predicting the P/R (progression or recurrence)	78 (33 with P/R, 45 without P/R)	T1	LR	Tumors with log-transformed mean pixel intensity above the median showed a 0.44 HR for recurrence or progression compared to lower intensity tumors.
Zhang (83)	2020	Predicting the P/R (progression or recurrence)	50 (28 with P/R, 22 without P/R)	T2, T1CE	SVM	Radiomics analysis using preoperative CE T1WI and T2WI MRI could predict recurrence in NFPA and elevated radiomic scores correlated with reduced PFS times (p < 0.001).
Shen (84)	2023	Predicting residual tumor regrowth	114 (70 with residual regrowth, 34 with no residual regrowth)	T1, T2, T1CE	LR	T1WI&T2WI outperformed other combinations or single sequences, and the integration of preoperative and postoperative images proved more effective than using them individually.
Zhang (87)	2021	Predicting postoperative visual field recovery	131 (79 in the recovery group, 52 in the non-recovery group)	T2	SVM, RF, LDA	Three radiomic models based on preoperative T2WI all showed good performance, each with an AUC over 0.75.
Zhang (88)	2023	Predicting postoperative visual field recovery	130 (87 in the recovery group, 43 in the non-recovery group)	Preoperative and postoperative T2	LASSO	Postoperative changes in the optic chiasm were not significant predictors of visual outcomes, but delta-radiomics of the optic chiasm have prognostic value for visual recovery.
Fan (95)	2019	predicts radiotherapeutic response in acromegaly	57 (25 achieved remission, 32 did not)	T1, T1CE, T2	SVM	The clinical-radiomics model showed good discrimination abilities, achieving an AUC of 0.96, surpassing that of any single clinical feature or standalone radiomics model.
Park (89)	2021	Predicting dopamine agonist response in prolactinoma	177 (109 DA responders, 68 DA non-responders)	T2	RF, light gradient boosting machine, ET, quadratic discrimination analysis, linear discrimination analysis and soft voting ensemble classifier	The ensemble classifier (AUC=0.81) performs better than any other individual machine learning classifier. Two second-order features demonstrated significant correlation with baseline PRL levels.
Galm (90)	2020	predicting response to somatostatin receptor ligands (SRLs) in acromegaly	34 (17 SRL responders, 17 SRL non-responders)	T1	LR	MRI texture of T1WI can predict normalization of IGF-I with SRL therapy.
Kocak (91)	2019	predicting response to somatostatin receptor ligands in acromegaly	47 (24 SRL responders, 23 SRL non-responders)	T2	KNN, C4.5 algorithm	Texture analysis based on KNN outperformed T2-weighted relative signal intensity, as well as immunohistochemical granulation pattern assessment, in predictive accuracy.

## 4 Discussion

Since the radiomics research is grounded in imaging data, the investigation of optimal sequences is an ever-present issue. The research encompasses a broad range of sequences, including T1, T2, TICE, DWI, and DCE MRI. Studies have almost unanimously supported the superior value of multimodal imaging data over unimodal data (34, 55, 68, 79, 84), aligning with prior findings (96, 97). Undoubtedly, the value of multiple imaging modalities must be acknowledged, as they enable the full utilization of imaging data and maximize the information extracted from radiomic studies. However, an exception was noted in the work of Zhang et al. (52), who observed that T1CE imaging features did not provide an additional contribution to the prediction of NCAs when compared to T1 imaging features alone. It might be due to the update rates of the contrast agent among patients, which influences the MRI image signals and the texture features of T1CE images. Regarding the comparison of the single-sequence model, there is notable variability in the outcomes across different studies. Liu et al. (51) concluded that the most significant MR image data in a single parametric model for differential diagnosis is postcontrast T1 image followed by T2 image. Zhao et al. (34) arrived at a contrary conclusion, positing that T2WI exhibits superior performance in differentiating cystic-solid pituitary adenoma from craniopharyngioma. The work of Peng et al. (47) found that SF-1 family tumors demonstrated greater accuracy in T1WI and T2WI, while Pit-1 family tumors showed enhanced accuracy in TICE. These discrepancies highlight that the underlying mechanism warrants further investigation and elucidation. Simplifying and streamlining sequences minimizes input features and eliminates irrelevant or noisy data, enabling models to concentrate on critical information and reduce overfitting risks (98). Thus, it is both necessary and crucial for future studies to investigate and determine the most effective single imaging sequence. Some recommended exercising caution when utilizing radiomics derived from T1W and T2WI, as the robustness and reproducibility of features from certain specific sequences are more assured (99). The utility of specialized sequences like DWI and DCE-MRI has been investigated (44, 69). However, the use of these non-routine MRI sequences of PA patients presents challenges in data collection, which may impede their integration into daily clinical practice.

Another important aspect of radiomics is the methodological concerns in the practice of radiomics. Different delineations of the Region of Interest (ROI) can significantly influence the outcomes of imaging models, as the initial aim is to capture as much data as possible at the front end, thereby enriching the database for more valuable downstream analysis and mining. Zhang et al. (83) applied different ROI areas, including the original tumor mask and masks that included surrounding non-tumorous structures. Unexpectedly, the results revealed that the choice of ROI had a minimal impact on the outcomes. We consider a possible explanation: differing ROI delineations might generate similar or overlapping features, which could lead to extracted attributes that may not exhibit significant statistical variations. On the contrary, Park et al. (50) pointed out that imaging biomarkers derived from ROI, after excluding cystic/necrotic portions, tend to offer greater utility in predicting the granulation pattern of adenomas compared to biomarkers obtained from the entirety of the tumor. By considering subregions within the ROI, we can identify the tumor’s heterogeneity, providing more effective biologically-relevant imaging features (100). Classifier modeling can use artificial intelligence, machine learning, and statistical approaches (10). While only a fewer small-sample studies employed traditional statistical methods (44), machine learning approaches are better suited for handling high-dimensional data and have become the mainstream choice. In terms of specific algorithms, support vector machine (SVM), a supervised Learning method, is among the most widely used. Models based on SVM demonstrated commendable predictive capabilities in Wang’s research, achieving a performance AUC of 0.937 for the external dataset (56). In a comprehensive comparative evaluation of nine machine learning algorithms, LDA emerged as the superior algorithm, outperforming SVM (31). Ensemble algorithm, employing a soft voting strategy, typically delivers superior predictive performance by aggregating outputs from multiple weak classifiers. Park, Y. W. et al.’s (89) research corroborates the effectiveness of the ensemble algorithm, which shows the highest performance among the single classifiers. Recently, deep learning has gained much attention and four studies adopted this emerging approach (41, 46, 48, 101). With the advantage of the ability to automatically extract features, this method significantly reduces the workload of radiologists and minimizes interobserver variation (102). However, it comes with the issue of the ‘black box’ effect, where the decision-making process of the model is challenging to interpret or understand, potentially leading to ethical and accountability concerns.

Regarding the definition and interpretation of features chosen for model development, we have described some unique discoveries. Shape-related features are significantly correlated with aggressiveness, whether defined by invasion of surrounding structures or a high proliferation index (68–70). An exemplary characteristic is tumor sphericity, with aggressive tumors exhibiting low sphericity indicative of irregular growth. This can be readily interpreted in the context of malignant tumor growth patterns: the disorderly angiogenesis and molecular heterogeneity within tumor cells consequently lead to the complexity of tumor shape (103). Interestingly, Zhang et al. (52) discovered that NCAs exhibit lower sphericity compared to other subtypes, hinting at the potential invasiveness of NCAs. This finding aligns with clinical observations (104), indicating a correlation between the radiomics of NCAs and their aggressive behavior as noted in clinical settings. A similar observation was noted in SG adenomas (50, 51). Image texture, or termed first/second-order features, has been correlated with histopathological findings, such as tumor subtype (44, 45), granulation pattern (50, 51, 90) and consistency (75). Although previous studies have shown that DG somatotroph adenomas often exhibit hypo- or iso-intensity on T2WI during the visual qualitative evaluation (105, 106), texture analysis provide more detailed information, noting that SG adenomas exhibit a significantly higher 10th percentile of T2-weighted signal intensity compared to DG adenomas (50). Similarly, Liu et al. (51) noted that DG and SG adenomas show significant differences in first-order features such as maximum value, median value, and 90th percentile voxel intensity. This is noteworthy because DG adenomas are characterized by an abundance of GH vesicles, a notably active Golgi apparatus, and a multitude of secretory granules, which could lead to a high concentration of intragranular proteins (107, 108). This composition is likely to shorten the T2 relaxation time, resulting in T2 hypo-intensity. Galm, B. P. et al. (90) proposed that texture features may be the radiological equivalent of granulation density. Higher-order features are typically extracted through mathematical transformations of the image, such as wavelet transforms and Fourier transforms (109). These features can reveal deeper information in images, enhancing the accuracy of predictions. Unfortunately, the biological interpretability of most high-order features is poor. It should be emphasized that radiomic analyses are tools for identifying correlations, rather than establishing causality (10). If a feature is only correlated with the outcome variable without a clear understanding of the biological or clinical mechanisms behind this correlation, then the practical utility and credibility of this feature may be called into question.

The advantages of radiomics extend beyond its non-invasive nature and precise identification capabilities. Radiomics offers a holistic approach in the context of tumor heterogeneity, where a biopsy can only provide specific analysis of surgically obtained localized tissue samples, potentially leading to misinterpretation due to the varying immunohistochemical profiles across different tumor regions. In contrast, radiomics allows for a comprehensive and intuitive evaluation of the entire tumor mass, facilitating a more accurate correlation with pathological findings (100). This is particularly relevant in pituitary adenomas, where the presence of adenoma with multiple staining positive results is not uncommon (110, 111), such as in plurihormonal adenomas or mixed tumors (112, 113). Additionally, the limitations of surgical resection, especially in endoscopic procedures, may impede the acquisition of complete tumor tissue samples.

## 5 Limitations

While highly promising, a major challenge limiting radiomics clinical translation is lack of generalization due to variation in acquisition parameters and radiomics approaches, which ultimately results in the difficulty of reproducibly acquiring stable radiomic features. Multiple studies have demonstrated parameters in MRI acquisition can affect radiomics analysis, including image contrast, slice thickness, magnetic strength and scanner platform (114, 115). Variation in the segmentation of ROI is another critical factor affecting the feature attributes. Fully automatic image segmentation can help to reduce the influence of operator-dependent bias on radiomic features (99). Additionally, the statistical reliability of features is also strongly affected by different software or even different versions of the same software (116, 117). In short, while there are existing guidelines on radiomic data acquisition and analysis (117–119), a standardized overall workflow of radiomics is not available yet, making the reproducibility and repeatability of features almost impossible. As with any biomarker, only stable and reproducible radiomic features can be applied in the complex and variable clinical environment (120). However, the lack of standardized experimental protocols prevents meaningful comparison of results between studies, making it difficult to discuss the merits of features or models. Each study ends up “speaking its own language,” with limited opportunities for cross-comparison or validation, hindering the development of consensus that could inform clinical practice globally.

For research of radiomics in pituitary tumors, A. Saha et al. (121) conducted a literature review covering the years 2009 to 2019, during which they found that only 11 of the studies were based on MR. As of September 2023, we have selected a total of 39 articles for our review. The increasing number of studies is commendable, but the issue of research quality cannot be overlooked. A systematic review from 2022 highlighted that the reporting quality of radiomics studies on pituitary adenoma is inadequate, with an average radiomics quality score of 26.6 (122). As they say, external validation is necessary. The majority of studies are retrospective and conducted at single centers with inevitable selection bias. Our literature review revealed that only a few studies included external validation sets (41, 44, 65), and even fewer, just one study (44), conducted prospective validation. High-quality and large-scale datasets are scarce, and small sample sizes and single-center studies often result in limited data representativeness. The lack of evidence-based medical proof makes clinicians more cautious about adopting radiomics.

The intrinsic characteristics of pituitary tumors also pose practical challenges, as they exhibit a wide variety of subtypes with significant epidemiological differences (7, 123, 124). A reasonable rule is that models based on binary classifiers, a minimum of 10 patients is required for each feature (10). Clearly, for low-prevalence or low-incidence subtypes, it is challenging for some studies to obtain a sufficient number of target cases to support the data volume required for radiomics, and the included cohorts may not represent the true proportion of the target patient population. Carlo et al. (45) implemented the synthetic minority over-sampling technique (SMOTE) to artificially expand the dataset size, but the efficacy of this approach in terms of performance on real-world data remains a subject for further investigation. Promoting large-scale cohort studies spanning various institutions, or facilitating the sharing of case data, may provide a viable solution to this impediment. Another issue is that the big data techniques applied in radiomics run counter to the concept of precision medicine, the former focus on pattern extraction from numerous cases and may potentially overlook individual variances. This issue is particularly pronounced when dealing with heterogeneous groups like those with PAs of different hormonal types. Integrating multi-omics data, including genomics, proteomics, and even laboratory test results, allows for a comprehensive and accurate assessment of patient conditions. Currently, incorporating well-established clinical variables, such as hormone levels, into predictive models in the form of nomograms has proven to be advantageous (34, 125).

It is important to note that radiomics should be considered an adjunctive tool rather than an independent diagnostic algorithm (126). In certain aspects, the necessity of radiomics is questionable, as the efficiency and simplicity of laboratory tests make the diagnosis, differentiation, and prognostic assessment of functional pituitary adenomas through radiomics seems redundant. Interestingly, in the 5th Edition of the WHO Classification of Endocrine and Neuroendocrine Tumors, tumor classification now relies on transcription factors, hormones, and other biomarkers, moving beyond the conventional hormone-based classification (3). When tumor tissue is unavailable, elucidating the relationship between radiomic features and histopathology to predict transcription factors or other biomarkers offers a potentially viable approach. However, the radiomic model of Baysal B. et al. demonstrates the least effective recognition of PHA (48), with an AUC of 0.74, which is considered acceptable but far from excellent (127). This highlights a current limitation in the application of radiomics for guiding pathological analysis. Nonetheless, it represents a promising direction for future development.

Additionally, there are several practical considerations for the real-world application of radiomics. Radiomics can leverage imaging data from routine clinical workflows without adding extra burden to patients, as MRI is indispensable (7). However, for healthcare institutions, high-resolution medical images and extensive feature extraction analysis demand robust computing power and storage capacity, which brings a substantial economic burden (128). Radiomics models access extensive patient data, raising concerns about data security and patient privacy. The complexity of computer technology and deep learning involved further discourages clinical and radiology practitioners from adopting this new technology. While end-to-end automated tools, such as AI models generating radiology reports directly, can lower the usage threshold for clinicians, this brings us to an ethical issue: who is responsible for the results (129)? The legal and ethical frameworks concerning the responsibility for artificial intelligence need urgent refinement.

## 6 Conclusion

In recent years, radiomics has made significant progress and has demonstrated potential applications in differential diagnosis, subtype identification, consistency evaluation, invasiveness assessment, and treatment response of pituitary adenomas. It has also established connections between radiological features and biological findings. However, the absence of standardized protocols and the need for enhancement in radiomic features, combined with the generally poor quality of existing studies, present significant challenges for clinical application. This technology holds more promise for non-functional pituitary adenomas. Moreover, given the complex nature of pituitary adenomas, it is clear that the clinical translation of radiomics in this field still has a considerable way to go.
